# Characteristics of HIV-1 Discordant Couples Enrolled in a Trial of HSV-2 Suppression to Reduce HIV-1 Transmission: The Partners Study

**DOI:** 10.1371/journal.pone.0005272

**Published:** 2009-04-30

**Authors:** Jairam R. Lingappa, Erin Kahle, Nelly Mugo, Andrew Mujugira, Amalia Magaret, Jared Baeten, Elizabeth A. Bukusi, Craig R. Cohen, Elly Katabira, Allan Ronald, James Kiarie, Carey Farquhar, Grace John Stewart, Joseph Makhema, M. Essex, Edwin Were, Kenneth Fife, Guy deBruyn, Glenda Gray, James McIntyre, Rachel Manongi, Saidi Kapiga, David Coetzee, Susan Allen, Mubiana Inambao, Kayitesi Kayitenkore, Etienne Karita, William Kanweka, Sinead Delany, Helen Rees, Bellington Vwalika, Robert W. Coombs, Rhoda Morrow, William Whittington, Lawrence Corey, Anna Wald, Connie Celum, for the Partners HSV-2/HIV-1 Transmission Study Team

**Affiliations:** 1 Department of Global Health, University of Washington, Seattle, Washington, United States of America; 2 Department of Medicine, University of Washington, Seattle, Washington, United States of America; 3 Department of Pediatrics, University of Washington, Seattle, Washington, United States of America; 4 Department of Obstetrics & Gynecology, University of Nairobi & Kenyatta National Hospital, Nairobi, Kenya; 5 Department of Laboratory Medicine, University of Washington, Seattle, Washington, United States of America; 6 Vaccine and Infectious Disease Institute, Fred Hutchinson Cancer Institute, Seattle, Washington, United States of America; 7 Center for Microbiology Research, Kenya Medical Research Institute, Nairobi, Kenya; 8 Department of Obstetrics, Gynecology and Reproductive Sciences, University of California San Francisco, San Francisco, California, United States of America; 9 Infectious Disease Institute, Makerere University, Kampala, Uganda; 10 University of Manitoba, Winnipeg, Manitoba, Canada; 11 Department of Epidemiology, University of Washington, Seattle, Washington, United States of America; 12 Botswana-Harvard Partnership, Gaborone, Botswana; 13 Department of Immunology & Infectious Diseases, Harvard School of Public Health, Harvard University, Boston, Massachusetts, United States of America; 14 Department of Reproductive Health, Moi University, Eldoret, Kenya; 15 Department of Medicine, Indiana University, Indianapolis, Indiana, United States of America; 16 Perinatal HIV Research Unit, University of Witwatersrand, Johannesburg, Republic of South Africa; 17 Kilimanjaro Christian Medical Centre, Moshi, Tanzania; 18 Department of Epidemiology & Population Health, London School of Hygiene and Tropical Medicine, London, England; 19 Infectious Disease Epidemiology Unit, University of Cape Town, Cape Town, Republic of South Africa; 20 Rwanda-Zambia HIV Research Group (RZHRG) and Emory University, Rollins School of Public Health, Atlanta, Georgia, United States of America; 21 Rwanda-Zambia HIV Research Group (RZHRG), Ndola, Zambia; 22 Rwanda-Zambia HIV Research Group (RZHRG), Kigali, Rwanda; 23 Rwanda-Zambia HIV Research Group (RZHRG), Kitwe, Zambia; 24 Reproductive Health & HIV Research Unit, University of Witwatersrand, Johannesburg, Republic of South Africa; 25 Rwanda-Zambia HIV Research Group (RZHRG), Lusaka, Zambia; University of New South Wales, Australia

## Abstract

**Background:**

The Partners HSV-2/HIV-1 Transmission Study (Partners Study) is a phase III, placebo-controlled trial of daily acyclovir for genital herpes (HSV-2) suppression among HIV-1/HSV-2 co-infected persons to reduce HIV-1 transmission to their HIV-1 susceptible partners, which requires recruitment of HIV-1 serodiscordant heterosexual couples. We describe the baseline characteristics of this cohort.

**Methods:**

HIV-1 serodiscordant heterosexual couples, in which the HIV-1 infected partner was HSV-2 seropositive, had a CD4 count ≥250 cells/mcL and was not on antiretroviral therapy, were enrolled at 14 sites in East and Southern Africa. Demographic, behavioral, clinical and laboratory characteristics were assessed.

**Results:**

Of the 3408 HIV-1 serodiscordant couples enrolled, 67% of the HIV-1 infected partners were women. Couples had cohabitated for a median of 5 years (range 2–9) with 28% reporting unprotected sex in the month prior to enrollment. Among HIV-1 susceptible participants, 86% of women and 59% of men were HSV-2 seropositive. Other laboratory-diagnosed sexually transmitted infections were uncommon (<5%), except for *Trichomonas vaginalis* in 14% of HIV-1 infected women. Median baseline CD4 count for HIV-1 infected participants was 462cells/mcL and median HIV-1 plasma RNA was 4.2 log_10_ copies/mL. After adjusting for age and African region, correlates of HIV-1 RNA level included male gender (+0.24 log_10_ copies/mL; p<0.001) and CD4 count (−0.25 and −0.55 log_10_ copies/mL for CD4 350–499 and >500 relative to <350, respectively, p<0.001).

**Conclusions:**

The Partners Study successfully enrolled a cohort of 3408 heterosexual HIV-1 serodiscordant couples in Africa at high risk for HIV-1 transmission. Follow-up of this cohort will evaluate the efficacy of acyclovir for HSV-2 suppression in preventing HIV-1 transmission and provide insights into biological and behavioral factors determining heterosexual HIV-1 transmission.

**Trial Registration:**

ClinicalTrials.gov NCT00194519

## Introduction

Worldwide, herpes simplex virus type 2 (HSV-2) is the most common cause of genital ulcer disease (GUD), with seroprevalence ranging from 22% to 60% in HIV-1 susceptible persons and often >70% among HIV-1 infected populations [1]. Numerous epidemiological studies have indicated that HSV-2 and HIV-1 interact to promote acquisition and transmission of each virus. Asymptomatic and symptomatic HSV-2 reactivation is associated with increased HIV-1 levels in blood [2], [3], cervical [4], [5] and rectal secretions [6], and semen [7]. Furthermore, observational studies in Rakai, Uganda found self-reported GUD in the prior 10 months among HIV-1 infected persons was associated with a 4-fold increased per contact risk of HIV-1 transmission [8]. Mathematical modeling studies suggest that the population-attributable risk of HSV-2 for HIV-1 acquisition in areas of high HSV-2 prevalence, such as sub-Saharan Africa, is between 25% and 50% [9], [10], [11].

These findings raise the possibility that HSV-2 suppression, using standard doses of acyclovir [12] in HSV-2/HIV-1 co-infected individuals, could reduce HIV-1 transmission to their HIV-1 susceptible partners. A direct test of this hypothesis requires a clinical trial enrolling HIV-1 serodiscordant couples in which the HIV-1 infected partner is co-infected with HSV-2 and randomized to daily antiviral therapy for HSV-2 suppression or matching placebo and the outcome (HIV-1 transmission) is measured in the HIV-1 susceptible partner. However, an efficacy trial requires recruitment of greater than 3000 HIV-1 serodiscordant couples if the annual HIV-1 transmission rate is 4%. Numerous challenges exist for this type of study design including recruitment and retention of both partners in HIV-1 serodiscordant couples, reluctance of individuals, particularly men, and couples to be tested for HIV-1, the need for couples to disclose their HIV-1 status to partners, and skepticism, among counselors and in communities where couples were recruited, about the possibility of HIV-1 serodiscordance in the context of a committed partnership.

The Partners in Prevention HSV-2/HIV-1 Transmission Study (“Partners Study”) is a randomized, placebo controlled clinical trial of acyclovir for HSV-2 suppression to reduce HIV-1 transmission in HIV-1 serodiscordant couples in multiple sites in East and Southern Africa. Enrollment into the clinical trial began in November 2004 and was completed in April 2007. Here we describe baseline characteristics of this unique cohort of HIV-1 serodiscordant couples recruited into this trial of HSV-2 suppression for HIV-1 prevention.

## Methods

### Study design

HIV-1 discordant couples for the Partners Study were recruited from seven sites in East Africa, and seven sites in Southern Africa (Table 1). The HIV-1 infected partners were randomly assigned to receive acyclovir (400 mg orally twice daily; Ranbaxy Inc., Haryana, India) or matching placebo. The primary clinical trial endpoint is seroconversion in the initially HIV-1 susceptible partner. We based sample size calculations for this study on an expected overall annual HIV-1 incidence rate of 4% in the placebo arm. Although estimates of incidence in HIV-1 discordant couples vary widely, the 4% incidence assumption was derived from prospectively-followed African HIV-1 serodiscordant couples demonstrating an annual HIV-1 incidence of 6.2–8.2% [13], combined with the assumption that regular HIV-1 prevention counseling at scheduled visits (monthly for the HIV-1 infected partner and quarterly for the HIV-1 uninfected partner) would further reduce incidence.

**Table 1:** HIV-1 and CD4 Diagnostic Assays Used for Study Eligibility.

Assay Type		Standard Test Kits/Instrument	Alternative Test Kits/Instrument*			
**HIV-1 paired rapid tests** †		Determine HIV 1/2 (Abbott Labs)	–	–	–	–
		Uni-Gold Recombigen HIV (Trinity Biotech PLC)	Capillus HIV-1/HIV-2 (Trinity Biotech PLC)	–	–	–
**HIV-1 EIA** ‡	Primary	Vironostika Uni-Form II Ag/Ab(bioMerieux SA)	Vironostika HIV-1 plus O (bioMerieux SA)	Vironostika HIV Uni-Form II Ag/Ab (bioMerieux SA)	Abbott AxSYM HIV Ag/Ab Combo (Abbott Labs)	Murex HIV-1.2.0 ELISA (Abbott Labs)
	Secondary	Enzygnost HIV Integral (Dade Behring)	Enzygnost (3^rd^ gen) (Dade Behring)	Murex HIV Ag-Ab combination assay (Murex Biotech)	Biorad HIV type 1 (Viral Lysate and Ecoli Recombinant Antigen)	Ortho HIV-1/2 Ab Capture Test System (Ortho)
**CD4 Count**	Instrument	FACSCount	Epics XL-MCL Flow Cytometer	–	–	–
	Method/Reagents	TruCount/Becton Dickenson	PLG CD4/Becton-Coulter	–	–	–

* All assays were required to pass external quality assessment prior to implementation for study-related testing and were required to undergo ongoing external quality assessment for the duration of the study.

† Paired HIV-1 rapid tests run in parallel.

‡ Confirmation of rapid test positive results performed with 2 wells on primary EIA kit. If initial EIA results were discrepant, the assay was repeated with one well each using the primary and secondary EIA kits.

### Enrollment

Recruitment of HIV-1 serodiscordant heterosexual couples for this trial has been described [14]. Briefly, counselors from Partners Study clinics and affiliated community voluntary counseling and testing (VCT) centers were trained in specialized skills in risk assessment, pre- and post-HIV test couples counseling through a training curriculum for a Couples HIV-1 Counseling and Testing (CHCT) developed in a collaborative effort between Rwanda Zambia HIV Research Group (Dr. Susan Allen), Liverpool VCT, Kenya (Dr. Miriam Taegtmeyer), and US Centers for Disease Control and Prevention [15]. Couples who tested as HIV-1 serodiscordant through community VCT or at a Partners study clinic were offered information about the study and referred for study screening.

HIV-1 serodiscordant couples were eligible for enrollment if they were sexually active (defined as vaginal or anal intercourse at least three times in the last three months), able to provide independent informed consent for participation in the study, planned to remain in the relationship for the duration of study follow-up (maximum 24 months), and provided locator information. Both partners consented to disclose HIV-1 test results to one another. Couples were ineligible if either partner was co-enrolled in another HIV-1 prevention or treatment trial, if the HIV-1 infected woman was pregnant based on self-report or urine testing at enrollment, and if the HIV-1 infected partners were excluded if they had a history of AIDS-defining diagnoses by WHO criteria or were taking antiretroviral therapy (ART) at the time of enrollment.

Laboratory eligibility requirements for enrollment of the HIV-1 infected partners included local site testing showing positive HIV-1 enzyme immunoassay (EIA), positive Focus HSV-2 EIA (see below), and CD4 cell count ≥250 cells/mcL. 

The only laboratory eligibility requirement for HIV-1 susceptible partners was HIV-1 EIA negative results.

Although condom use was not an eligibility requirement, all study participants were provided information and individuals and couples counseling regarding HIV-1 risk reduction, study drug adherence and family planning, free condoms, and access to ART if clinically indicated, based on clinical symptoms, CD4 testing, and in accordance with national guidelines.

### Data collection

Demographic, behavioral and clinical data were collected from enrolled participants through interviewer-administered questions using forms translated into local languages and back translated to ensure appropriate content. Data forms were scanned and entered using intelligent character recognition (ICR) DataFax software (DataFax, ver 3.7-004, Clinical DataFax Systems Inc., Hamilton, Ontario, Canada) and centrally double-verified by independent data technicians.

### Laboratory testing

Study clinics screened potential study participants for HIV-1 using paired commercial HIV-1 rapid tests run in parallel. HIV-1 3^rd^ or 4^th^ generation enzyme immunoassays (EIA) were used to confirm concordant positive paired rapid-assay reactive results at the local laboratories (Table 1). Couples in which either partner had discrepant results from their paired HIV-1 rapid assays were excluded from study enrollment.

HSV-2 serostatus was assessed at the local laboratories using HerpeSelect HSV IgG ELISA (“Focus”; Focus Technologies, Cypress, CA, USA). Samples with a Focus index value ≥3.5, were considered HSV-2 seropositive. In addition, HIV-1 infected participants with a Focus index value between 1.1 and 3.4, potentially indicative of HSV-2 seroconversion, had serum sent to the University of Washington; those which were positive by Western blot (WB) were also considered HSV-2 seropositive [16]. The protocol required HIV-1 infected partners to be screened for HSV-2 seropositivity by Focus EIA at the local study site, whereas there was no eligibility requirement for HSV-2 serostatus of HIV-1 negative partners. For this analysis, HSV-2 serostatus of HIV-1 negative enrolled partners is based on batched HSV-2 WB confirmation performed at the University of Washington.

CD4 testing of HIV-1 infected participants was performed at the study site using FacsCount or FacsCalibur Instrumentation (BD Biosciences, San Jose, USA). Plasma collected in acid citrate dextrose (ACD) from HIV-infected participants was assessed for HIV-1 RNA level through batch testing at the University of Washington using the 96-test COBAS AmpliPrep/COBAS TaqMan HIV-1 RNA assay, version 1.0, (Roche Diagnostics, Indianapolis, IN, USA). Plasma specimens were diluted 1∶5 dilution with 10× phosphate buffered saline (Invitrogen-GIBCO, Carlsbad, CA, USA) for the assay with a limit of quantification of 240 RNA copies/mL.

Sites performed syndromic diagnosis for the management of sexually transmitted infections. Syphilis serology was performed at all sites, using rapid plasma reagin (RPR) with confirmation by microhemagglutination test for *T. pallidum* (MHA-TP) at sites with that capacity. Participants with positive syphilis serologies received syphilis treatment per national guidelines. Cervical and urine samples were archived for batch testing for *Neisseria gonorrhoeae*, *Chlamydia trachomatis* and *Trichomonas vaginalis* at the University of Washington. Testing for *N. gonorrhoeae* and *C. trachomatis* was performed according to the manufacturer's protocol (APTIMA Combo 2 assay, Gen-Probe, San Diego, CA, USA). Testing for *T. vaginalis* (TV) was performed using a research assay with TV analyte–specific and APTIMA General Purpose Reagents [17]. Specimens yielding consistent relative light unit values >100,000 were considered positive for *T. vaginalis*.

### Evaluation of Quality Assessment (EQA)

All assays for study eligibility were performed at site local laboratories, specifically HIV-1 serology (rapid assays and EIA), HSV-2 Focus ELISA, and CD4 counts; and these were subjected to external EQA panels. An HSV-2 proficiency panel was developed at the University of Washington and implemented through Contract Laboratory Services (CLS, Dr. Wendy Stevens, Johannesburg, South Africa). Study-site laboratories and clinics were enrolled in EQA for HIV-1 serology through the National Health Laboratory Service National Institute of Communicable Diseases (NHLS/NICD) of South Africa. CD4 testing was assessed through panels distributed by WHO/QASI/NHLS system [18]. HIV-1 RNA testing at the University of Washington utilized proficiency-testing panels from the National Institutes of Health, Division of AIDS, Virology Quality Assurance Program.

### Statistical Analysis

Statistical comparisons were performed using SAS v.9.2 (SAS Institute, Cary, North Carolina). Differences by region, East versus Southern Africa, and by HIV-1 infection status were examined using Chi-square tests for dichotomous factors and linear regression for continuous measures. Predictors of baseline plasma HIV-1 RNA in HIV-1 infected participants were examined using linear regression. Plasma HIV-1 RNA in which the target was not detected or which were below limit of quantification (BLQ, 240 copies/mL) was set to 120 copies/mL.

### Ethics Statement

The Partners Study clinical trial protocol including forms for written informed consent were reviewed by ethics committees at the following institutions: South African sites: University of Witwatersrand, and University of Cape Town; Botswana site: Republic of Botswana Ministry of Health, and Harvard School of Public Health; Zambian sites: Tropical Disease Research Centre, Republic of Zambia National Ethics Committee, and Emory University; Rwandan site: Republic of Rwanda National Ethics Committee, Emory University; Tanzanian site: Kilimanjaro Christian Medical College, Harvard School of Public Health, and London School of Hygiene and Tropical Medicine; Ugandan site: Uganda National Ethics Committee; Kenyan sites: Kenyatta National Hospital, Moi University, Kenya Medical Research Institute, Indiana University, University of California San Francisco, and University of Washington.

## Results

**Figure 1:**
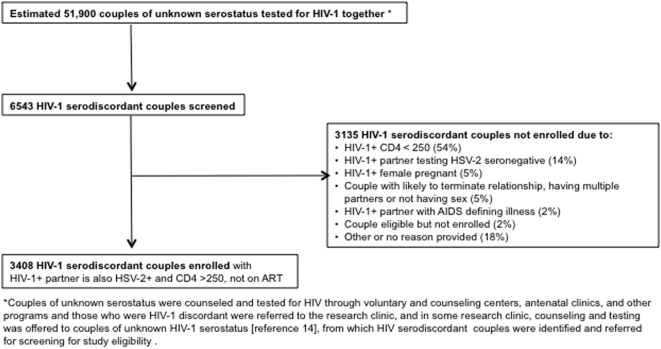
Prescreening, Screening and Enrollment Flow for HIV-1 serodiscordant couples in the Partners Study.

Figure 1 shows the prescreening, screening and enrollment flow for this study. The 14 study sites screened 6543 HIV-1 discordant couples for study eligibility, 3135 of which were identified as HIV-1 serodiscordant but not enrolled; the major reasons were for ineligibility were: HIV-1 infected partners with CD4 count <250 cells/mcL (54%), or HSV-2 seronegative (14%), or pregnant (5%). Thus, 3408 couples meeting the study eligibility requirements were enrolled with 66% from East African sites (Table 2). Enrolled participants were from diverse ethnic origins with 83% self-identifying into 38 distinct ethnic groups.

**Table 2:** Sites and total enrollment for Partners in Prevention HSV-2/HIV-1 Transmission Study (Partners Study).

Site Location	Couples Enrolled*
	Total
**East Africa**	**2250**
Kenya	
Kisumu	532
Nairobi	416
Eldoret	268
Thika	213
Rwanda	
Kigali	153
Tanzania	
Moshi	218
Uganda	
Kampala	450
**Southern Africa**	**1158**
Botswana	
Gaborone	325
South Africa	
Cape Town	196
Orange Farm	73
Soweto	240
Zambia	
Kitwe	91
Lusaka	72
Ndola	161
**All regions/sites**	**3408**

* Couples were enrolled at sites for time periods ranging from 5 to 30 months [14].

### Demographic and behavioral characteristics

Table 3 shows demographic and behavioral characteristics of enrolled couples. Of the 3408 HIV-1 infected participants, 16 enrolled two HIV-1 susceptible partners. HIV-1 infected study participants were predominantly female (67%) and married (76%) with a median age of 33 years (IQR 27–39). Conversely, the majority of HIV-1 susceptible participants were male with a median age of 35 years (IQR 28–41). Among couples that reported living together, the median duration of cohabitation was 5 years (IQR 3–10). Couples reported having a median of 2 children (IQR 1–4).

**Table 3:** Baseline Demographic and Behavioral Characteristics of Enrolled Couples by Gender of HIV-infected Partner.

Characteristic	Couples with HIV-infected women		Couples with HIV-infected men	
	HIV-infected female (#, %)	HIV susceptible male (#, %)*	HIV-infected male (#, %)	HIV susceptible female* (#, %)
**Total participants**	2299	2304	1109	1120
**Age (yrs)**				
Median (IQR)	30 (25–35)	35 (30–42)	37 (32–45)	31 (25–38)
18–24	476 (21%)	117 (5%)	36 (3%)	231 (21%)
25–34	1230 (54%)	982 (43%)	372 (34%))	502 (45%)
35–44	469 (20%)	752 (33%)	410 (37%)	284 (25%)
44+	122 (5%)	452 (20%)	288 (26%)	103 (9%)
**Partnership characteristics**				
Married to partner	1683 (73%)	–	897 (81%)	–
Living with partner	2037 (89%)	–	1035 (93%)	–
Yrs living w/partner (median, IQR)†	5 (2–9)	–	6 (3–13)	–
Number of children (median, IQR)†	2 (1–3)	–	3 (2–5)	–
Yrs education (median, IQR)	8 (6–10)	9 (7–12)	8 (7–11)	8 (6–10)
Any monthly income	564 (24%)	1377 (60%)	665 (60%)	289 (26%)
**Sex acts (in prior month)**				
Total sex acts (median, IQR)†	4 (2–8)	–	4 (2–8)	–
Unprotected sex acts (median, IQR)†	0 (0–1)	–	0 (0–1)	–
Couples reporting any unprotected sex acts	661 (29%)	–	311 (28%)	–
**Contraception**				
Condoms	1062 (46%)	–	–	490 (44%)
Oral	96 (4%)	–	–	47 (4%)
Injectable	326 (14%)	–	–	123 (11%)
Other (IUD, sterilization)	188 (8%)	–	–	135 (12%)
None	727 (32%)	–	–	372 (33%)

* Sixteen enrolled couples included one HIV-1 infected participant and two HIV-1 uninfected partners.

† Only data collected from the HIV-1 infected partner was for these couples-level characteristics.

At enrollment, couples reported a median of 4 sex acts (IQR 2–8) (vaginal and/or anal intercourse) in the prior month, and 28% reported at least one episode of sex without a condom in the past month. Among all HIV-1 infected and HIV-1 susceptible men, 5% reported sex acts with partners other than their enrolled partner in the month prior to enrollment, compared to <2% of women. Nearly half of women (46% of HIV-1 infected and 44% of HIV-1 susceptible) reported using only condoms for contraception, and one-third of women (32% and 33% of HIV-1 infected and HIV-1 susceptible women, respectively) reported not using any form of contraception.

### Clinical characteristics

A history of GUD (a history of genital ulcers or diagnosis of GUD in the previous three months) was reported in 6% and 8% of HIV-1 infected men and women, respectively; and 2% and 5% of HIV-1 susceptible men and women, respectively (Table 4). Genital ulcers were detected on physical exam at enrollment in 3% and 4% of HIV-1 infected men and women, respectively, compared to 1% of HIV-1 susceptible partners. Male circumcision, based on genital exam, was more prevalent in HIV-1 susceptible compared to HIV-1 infected men (55% versus 34%, respectively; p<0.001). East African men were more likely to be circumcised than Southern African men both among HIV-1 uninfected men (64% versus 37%, p<0.001) and HIV-1 infected men (39% versus 23%, p<0.001).

**Table 4:** Baseline Clinical and Laboratory Characteristics of Enrolled Couples by Gender of HIV-1 Infected Partner.

Characteristic	Couples with HIV-infected women		Couples with HIV-infected men	
	HIV-infected female (#, %)	HIV susceptible male (#, %)*	HIV-infected male (#, %)	HIV susceptible female* (#, %)
**Symptoms of GUD (previous 3 months)**	174 (8%)	46 (2%)	63 (6%)	54 (5%)
**Syndromic STI diagnosis (previous 3 months)**				
Vaginitis	251 (11%)	–	–	51 (5%)
Cervicitis	28 (1%)	–	–	9 (1%)
PID	50 (2%)	–	–	19 (2%)
Urethritis	65 (3%)	50 (2%)	25 (2%)	17 (2%)
**Physical exam findings**				
Cervical tenderness	59 (3%)	–	–	20 (2%)
Cervical mucopus	56 (2%)	–	–	27(2%)
Circumcision (males only)	–	1255 (55%)	381 (34%)	–
Genital Ulcers	92 (4%)	31 (1%)	34 (3%)	14 (1%)
**CD4 count** (cells/mcL) (median, IQR)	483 (355–665)	–	424 (334–571)	–
**HIV-1 Plasma RNA**				
Log_10_ Median (IQR)	4.1 (3.5–4.6)	–	4.4 (3.8–4.9)	–
BLQ**	288 (14%)	–	67 (6%)	–
<2000 copies/mL	335 (15%)	–	108 (10%)	–
2000–10,000 copies/mL	572 (25%)	–	221 (20%)	–
>10,000 copies/mL	1078 (47%)	–	702 (63%)	–
**HSV-2 Western Blot**				
HSV-2 seropositive	*	1362 (59%)	*	954 (85%)
**Sexually Transmitted Infections**				
*N. gonorrhoeae* positive (TMA)	40 (2%)	11 (1%)	9 (1%)	12 (1%)
*C. trachomatis* positive (TMA)	54 (2%)	64 (3.0%)	14 (1%)	19 (2%)
*T. vaginalis* positive (TMA)	367 (16%)	157 (7%)	52 (5%)	123 (11%)
Positive RPR	140 (6%)	107 (5%)	61 (6%)	43 (4%)
RPR titer (>1∶8)	94 (4%)	62 (3%)	41 (4%)	23 (2%)

* All HIV-1 infected participants were HSV-2 seropositive at enrollment based on Focus EIA (index value >3.4) testing at the study site.

** Below Limit of Quantitation.

### Laboratory characteristics

Table 4 shows local and central laboratory data for enrolled couples. Overall 68% of HIV-1 susceptible participants were identified as HSV-2 seropositive with higher prevalence in women compared to men (85% versus 59%, p<0.001; Table 4). Nucleic acid testing for *N. gonorrhoeae* and *C. trachomatis* in both HIV-1 infected and HIV-1 susceptible was positive in ≤2% of individuals; *T. vaginalis* was more prevalent than other STIs and more commonly detected in women (11% in HIV-1 susceptible and 16% in HIV-1 infected women) than in men (7% in HIV-1 susceptible and 5% in HIV-1 infected men). Positive RPR tests for syphilis ranged from 4 to 6%; 3% of all participants had an RPR titer >1∶8 indicative of possible early syphilis.

Median CD4 count at enrollment among HIV-1 infected women and men was 483 (IQR 355–665) and 424 (IQR 334–571) cells/mcL, respectively. The median baseline plasma HIV-1 RNA for HIV-1 infected participants was 4.2 (IQR 3.63–4.76) log_10_ copies/mL (equivalent to 15,800 copies/mL); with 26% having HIV-1 RNA <2000 copies/mL at enrollment and 24% having >50,000 copies/mL. In univariate analysis, plasma HIV-1 RNA did not vary by East or Southern African region (p = 0.15), but did vary by age, gender, and CD4 count. In a multivariable regression model controlling for age and region, CD4 count and gender remained significantly associated with plasma HIV-1 RNA: men had plasma HIV-1 RNA 0.24 log_10_ copies/mL higher than women; and persons with CD4 counts ≥500 cells/mcL and 350–499 cells/mcL had 0.55 log_10_ and 0.25 log_10_ copies/mL lower plasma HIV-1 RNA, respectively, than persons with CD4 counts 250–350 cells/mcL (Table 5).

**Table 5:** Determinants of Baseline Plasma RNA in Partners Study HIV-1 Discordant Couples.

Factor	HIV-1 RNA (mean, log_10_)	Univariate Analysis (P-value)	Multivariable Analysis	
			Change in HIV-1 RNA vs Referent (95% CI)	P-value
**Age**				
18–24	4.04 (3.96, 4.12)	Referent	—	Referent
25–34	4.01 (3.96, 4.06)	0.52	−0.11 (−0.2, 0.02)	0.01
35–44	4.14 (4.08, 4.20)	0.05	−0.07 (−0.17, 0.03)	0.17
44+	4.29 (4.21, 4.38)	<0.0001	−0.02 (−0.14, 0.1)*	0.76
**Gender**				
Women	3.98 (3.94, 4.02)	Referent	—	Referent
Men	4.29 (4.24, 4.34)	<0.0001	0.24 (0.3, 0.18)	<0.0001
**CD4 count**				
250–349 cells/mm^3^	4.41 (4.35, 4.47)	Referent	—	Referent
350–499 cells/mm^3^	4.16 (4.11, 4.21)	<0.0001	−0.25 (−0.33, −0.17)	<0.0001
500+ cells/mm^3^	3.83 (3.79, 3.88)	<0.0001	−0.55 (−0.63, −0.48)	<0.0001
**Region**				
Southern Africa	4.05 (4.00, 4.10)	Referent	—	Referent
East Africa	4.10 (4.06, 4.14)	0.16	0.07 (0.01, 0.14)	0.02

* The impact of older age on HIV-1 RNA is likely accounted for by interaction between age and gender in the multivariable analysis.

## Discussion

The 3408 HIV-1 serodiscordant couples (6832 individuals) enrolled in the Partners Study, is the largest, most ethnically and geographically diverse cohort of HIV-1 serodiscordant couples ever recruited into an HIV-1 prevention clinical trial.

The HIV-1 serodiscordant couples study design for this clinical trial was dictated by the need to directly assess whether HSV-2 suppression in an HIV-1 infected partner reduced the risk of HIV-1 transmission to their sexual partner. In order to identify these 3408 couples, approximately 51,900 couples of unknown HIV-1 serostatus were tested for HIV-1 and 6543 HIV-1 serodiscordant couples screened for study eligibility. Thus, this trial required multiple sites and intensive community outreach efforts to promote couples counseling with strong linkages with HIV-1 voluntary testing and counseling centers, antenatal clinics, and other clinical and community organizations.

The demographic, clinical and laboratory characteristics of this large and diverse cohort of HIV-1 serodiscordant couples suggest that there are subgroups with distinct HIV-1 transmission risk profiles. First, the median plasma HIV-1 RNA for HIV-1 infected partners in this cohort was 4.2 log_10_ copies/mL, with a quarter of the cohort having plasma RNA ≥4.7 log_10_ copies/mL (50,000 copies/mL). The observational study of monogamous HIV-1 serodiscordant couples from Rakai, Uganda identified plasma HIV-1 RNA as the strongest predictor of HIV-1 transmission; persons with HIV-1 RNA ≥50,000 copies/mL had a 12-fold increased risk of HIV-1 transmission compared to those with HIV-1 RNA ≤3500 copies/mL [19]. Second, nearly one-third of couples reported at least one episode of unprotected sexual intercourse in the prior month. Finally, the majority of HIV-1 susceptible participants were HSV-2 seropositive (68%) with higher prevalence in women as has been reported in other populations [20]. Previous longitudinal studies have found HSV-2 infection in HIV-1 susceptible people is associated with 1.8 to 3.1-fold increased risk of HIV-1 acquisition [1], [9], [21], [22].

Notably, a quarter (26%) of HIV-1 infected participants had HIV-1 RNA <2000 copies/mL at baseline, which is associated with a reduced risk of HIV-1 transmission. The cutoff of HIV-1 RNA <2000 copies/mL has been used to identify viral “controllers”, i.e., ART-naïve individuals with who have maintained low HIV-1 RNA and who are at lower risk of HIV-1 transmission to their HIV-1 susceptible sex partners [19]. Prevalence of such viral controllers was less than 12% of HIV-1 infected partners identified through community surveillance in South Africa [23]. Use of ACD for plasma collection can underestimate plasma HIV-1 RNA by 0.11 log_10_ compared to EDTA plasma collected under ideal conditions [24]. Therefore, it is unlikely that choice of anticoagulant could account for the large proportion of viral controllers in this cohort. Further study will be conducted to determine whether low plasma HIV-1 RNA levels at enrollment is related to undisclosed ART use. The substantial minority of HIV-1 infected partners with low viral loads in this cohort may reflect a selection bias arising from recruitment of couples who had lived together for an average of five years without transmitting HIV-1. The higher prevalence of viral controllers among HIV-1 serodiscordant couples may present an opportunity to examine immunological and genetic factors associated with viral control in diverse African populations. More than half of the HIV-1 susceptible males in this cohort were circumcised (54%), representing a decreased risk of HIV-1 acquisition in those men who were circumcised, consistent with three recent trials, which have demonstrated that male circumcision lowers the risk of HIV-1 acquisition [25], [26], [27]. Finally, while laboratory-diagnosed sexually transmitted infections (*N. gonorrhoea*, *C. trachomatis* and *T. vaginalis*) have previously been identified as risk factors for HIV-1 acquisition and transmission [28], the prevalence of *N. gonorrhoeae* and *C. trachomatis* were very low in this cohort of stable couples with modestly higher prevalence of *T. vaginalis*. The role of these biologic co-factors and relative risk of HIV-1 transmission will be assessed after follow-up and unblinding is completed.

Recent clinical trials have shown that acyclovir suppressive therapy among high risk, HSV-2 seropositive/HIV-1 susceptible persons does not reduce their risk of acquiring HIV-1 [29], [30]. Those results may be understood in light of recent data documenting frequent subclinical HSV-2 reactivation in genital mucosa [31] with persistent host cellular immune responses, including HIV-1 susceptible CD4+ and CCR5+ T cells and plasmacytoid dendritic cells, present in HSV-2 lesions and persisting for weeks after HSV-2 treatment [32], [33], [34]. Thus, while daily antivirals in HSV-2 seropositive/HSV-1 uninfected persons reduce symptoms of genital ulcers and HSV-2 shedding, the persistence of HIV-1 susceptible cells associated with the host immune response to HSV-2 [33], [34] likely maintains a high risk of HIV-1 acquisition.

However, the lack of efficacy observed for HSV-2 suppression to reduce HIV-1 acquisition may not necessarily pertain to this trial of HSV-2 suppression to reduce HIV-1 transmission, as the biologic mechanisms for the two types of interactions differ. The effect of HSV-2 on HIV-1 transmission reflects the observation that HSV-2 infection in HIV-1/HSV-2 co-infected persons is associated with increased plasma [19] and genital HIV-1 RNA [35]. Although the biologic basis for these in vivo observations is still not completely understood, *in vitro* studies indicate that early and intermediate proteins associated with HSV viral replication facilitate HIV-1 replication through binding and transactivation of the long terminal repeat region of HIV-1, and genital herpes is associated with secretion of pro-inflammatory cytokines and activation of target cells for HIV-1 [36], [37], [38], [39]. Increased plasma or genital HIV-1 RNA associated with HSV-2 co-infection would result in exposure of an HIV-1 susceptible person to larger inoculums of HIV-1 during unprotected sexual intercourse with an HIV-1/HSV-2 co-infected person. Several proof-of-concept trials have shown that HSV-2 suppression with daily acyclovir or valacyclovir in HIV-1/HSV-2 co-infected persons reduces HIV-1 RNA by 0.26–0.53 log_10_ and cervical or rectal HIV-1 RNA by 0.16–0.35 log_10_ [6], [40], [41], [42], [43]. These reductions in plasma HIV-1 RNA may indicate that that HSV-2 suppression could also slow HIV-1 disease progression, as was suggested by a pooled analysis from the 1990s, which found that high-dose acyclovir, in combination with nucleoside ART, was associated with a survival benefit [44]. While these studies are encouraging, the outcome of the Partners Study is of critical importance to directly test the hypothesis that HSV-2 suppression will reduce HIV-1 transmission or HIV-1 disease progression.

Overall, the demographic and behavioral characteristics of this study cohort are similar to other cohorts of HIV-1 serodiscordant couples recruited for research studies in Africa [8], [45]. Notably however, the proportion of HIV-1 infected participants that were women (67%) is higher than the 30–50% previously reported [8], [45], [46], [47], [48]. The higher proportion of female HIV-1 infected partners among Partners couples enrolled in the Partners trial may derive from the preponderance of VCT and antenatal clinics for recruitment. Given lower HIV-1 testing rates among men and a tendency for African men to rely on their partners to be tested (assuming their partner's HIV serostatus is their own) [49], other recruitment strategies, such as home-based VCT [50], may be needed for future HIV-1 prevention trials to identify a higher proportion of HIV-1 serodiscordant couples with HIV-1 infected men.

HIV-1 serodiscordant couples are increasingly becoming a focus of HIV-1 prevention research particularly in Africa [51], [52], [53], [54], [55], in part due to the fact that HIV-1 discordance is common in many locations in Africa and that a substantial fraction of HIV-1 infections are transmitted in stable couples [14], [47]. Estimates of HIV-1 seroincidence in HIV-1 serodiscordant couples in sub-Saharan Africa vary greatly [55], but have been reported as high as 12% [19] to 22% [56] per year. HIV-1 discordant couples are clearly at high risk of HIV-1 transmission, and are a critical target for evaluation of new prevention interventions. Efforts to recruit HIV-1 discordant couples for research efforts helps build public health infrastructure for identifying such couples. The Partners Study has now clearly demonstrated that large numbers of HIV-1 discordant couples at high risk for HIV-1 transmission can be successfully recruited to an HIV-1 prevention clinical trial.
